# Non-Histone Lysine Modifications in Tumor Microenvironment: Mechanisms and Therapeutic Opportunities

**DOI:** 10.3390/ijms262211229

**Published:** 2025-11-20

**Authors:** Kai Sun, Shuying Xiao, Qibo Huang, Suhang Zhang, Qilin Li, Chuanyu Hu

**Affiliations:** 1 Department of Stomatology, Tongji Hospital, Tongji Medical College, Huazhong University of Science and Technology, Wuhan 430030, China; sunkai_tjh@163.com (K.S.); 17771594555@163.com (S.X.); npv942@163.com (S.Z.); 2 School of Stomatology, Tongji Medical College, Huazhong University of Science and Technology, Wuhan 430030, China; 3 Hubei Province Key Laboratory of Oral and Maxillofacial Development and Regeneration, Wuhan 430022, China; 4 Hepatic Surgery Center, Tongji Hospital, Tongji Medical College, Huazhong University of Science and Technology, Wuhan 430030, China; huangqbo_tjh@163.com

**Keywords:** post-translational modifications, non-histone lysine, tumorigenesis, tumor immune microenvironment

## Abstract

Post-translational modifications (PTMs) on protein lysine residues, including lactylation, methylation, acetylation, ubiquitination, and succinylation, serve as critical regulators in tumorigenesis and progression. Histone PTMs participate in tumor development by modulating chromatin structure and regulating gene expression. Notably, accumulating evidence reveals that PTMs target extensive non-histone substrates. These modifications occurring on non-histone proteins also contribute to tumor-associated biological processes. In this review, we systematically summarize the impact of non-histone PTMs on tumor and the tumor immune microenvironment (TIME). Additionally, we discuss crosstalk between distinct PTMs, which complicates the regulatory mechanisms of protein function. An in-depth research on PTMs in tumors holds new insights for exploring promising clinical therapeutic strategies.

## 1. Introduction

In eukaryotes, the complexity of the proteome surpasses genome by two to three orders of magnitude, a diversity mainly driven by post-translational modifications (PTMs) at one or more amino acid residues [1]. PTMs involve the enzymatic or non-enzymatic covalent attachment of specific chemical groups to amino acid side chains, leading to diverse modifications such as phosphorylation, methylation, acetylation, and glycosylation [2]. These modifications exert multifaceted effects on protein function, including regulating protein activity, altering subcellular localization, participating in signal transduction, and modulating protein–protein interactions [3,4]. By introducing dynamic chemical modifications, PTMs significantly expand the information capacity and regulatory dimensions of the genetic code, serving as central regulators in tumorigenesis and progression [4].

Among the 20 amino acids that constitute proteins, lysine is the most frequently subject to PTMs, playing extensive roles in cellular biological functions and disease progression [5,6]. Unlike histone PTMs, which rely on epigenetic mechanisms to regulate gene expression, non-histone PTMs exert multifaceted effects on protein function, including regulating protein activity, altering subcellular localization, participating in signal transduction, and modulating protein–protein interactions [7]. In this review, we will focus on emerging types of modifications and their roles in cancer, reassess the functions of non-histone lysine modifications, and discuss therapeutic strategies that target this chemical modification layer.

## 2. Non-Histone Lysine Modifications in Tumors

### 2.1. The Overview of Non-Histone Lysine Modifications in Tumors

**Figure 1:**
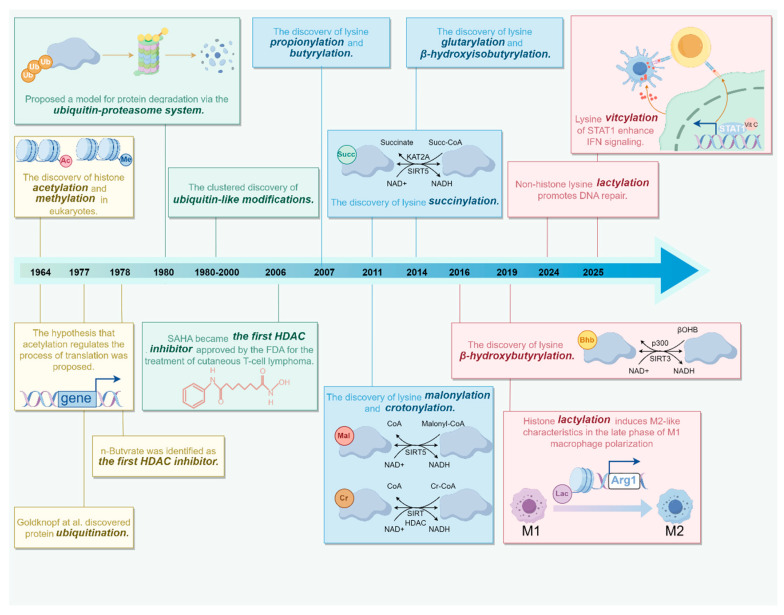
The timeline of lysine PTMs research. This figure summarizes key events in the history of lysine PTMs, starting from the first discovery of protein acetylation and methylation in eukaryotes in 1964. It includes the identification of novel PTMs, the emergence of new concepts, and advances in related technologies. Acetylation and methylation were discovered in 1964. Propionylation and butyrylation were discovered in 2007. Succinylation, malonylation, and crotonylation were discovered in 2011. Glutarylation and β-hydroxyisobutyrylation were discovered in 2014. β-hydroxybutyrylation was discovered in 2016. Lactylation was discovered in 2019. Vitcylation was discovered in 2025.

**Figure 2:**
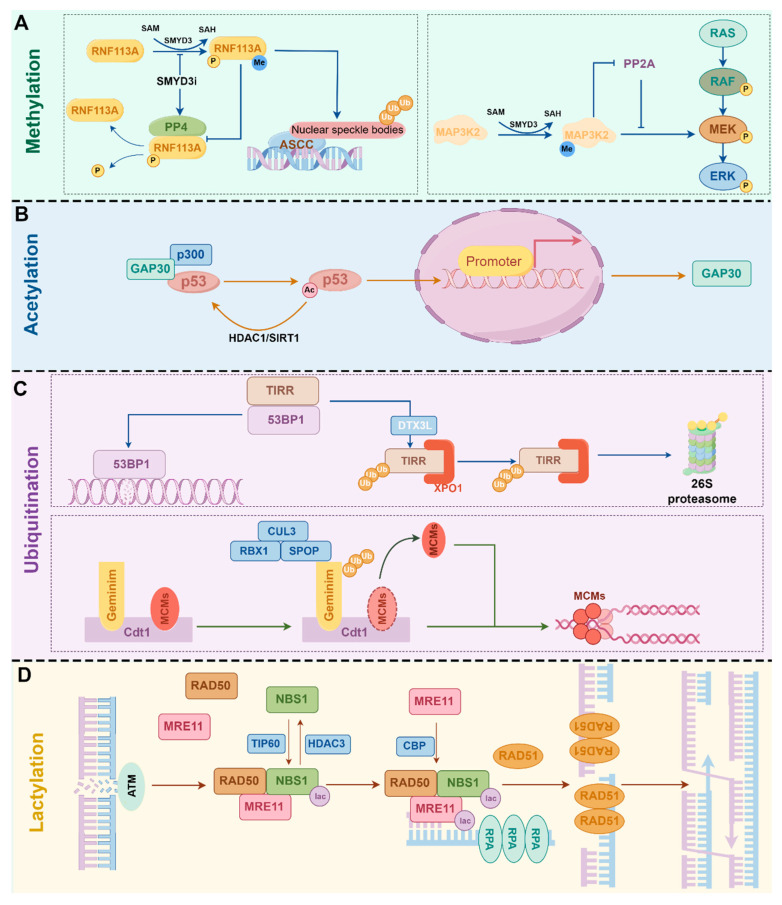
Non-histone lysine PTMs in tumors. (**A**) Methylation. methylation helps maintain the E3 ligase activity of RNF113A, which is crucial for the activation of ASCC in the alkylation damage response; methylation of MAP3K2 blocks its inhibition by PP2A, thereby driving Ras signaling. (**B**) Acetylation. p53 is acetylated by p300 and deacetylated by HDAC1 and SIRT1. Acetylation enhances the transcriptional activity of p53 and promotes the expression of its target genes. (**C**) Ubiquitination. DTX3L-mediated ubiquitination of TIRR promotes its degradation, leading to the release of 53BP1 and its subsequent binding to DSBs; ubiquitination of Geminin hinders Cdt1 from recruiting MCMs, thereby preventing DNA replication overactivation. (**D**) Lactylation. Lactylation of NBS1 promotes the formation of the MRN complex; subsequently, MRE11 lactylation enhances its binding to DSBs and facilitates DNA end resection. These effects collectively promote HR repair.

The discovery of histone methylation and acetylation in 1964 marked the first identification of PTM types and established a milestone in PTM research [8,9] (Figure 1). Lysine methylation is dynamically regulated by lysine methyltransferases (KMTs) and lysine demethylases (KDMs), with S-adenosylmethionine (SAM) serving as the primary methyl donor for this modification [10]. Based on structural domain characterization and substrate specificity, KMTs are classified into 12 distinct families, while KDMs are categorized into 7 families [11]. SMYD3-mediated methylation of MAP3K2 blocks its binding to the PP2A phosphatase complex, which is crucial for oncogenic Ras signaling [12]. SMYD3 also catalyzes RNF113A methylation, thereby maintaining the function of the activating signal cointegrator complex (ASCC) in dealkylation repair [13] (Figure 2A).

Acetylation occurs when an acetyl group is transferred enzymatically or non-enzymatically from acetyl-CoA to lysine residues [14]. Lysine acetyltransferases (KATs) catalyze the addition of acetyl groups to lysine, while lysine deacetylases (KDACs) remove them. To date, 22 distinct KATs have been identified, classified into three major families: GCN5, CBP/p300, and MYST. And 18 KDACs, categorized into two classes: Zn^2+^-dependent histone deacetylases (HDAC1-11) and NAD^+^-dependent deacetylases (SIRT1-7) [15]. ArhGAP30 acts as a “bridge” molecule by recruiting CBP/p300 to p53 and promoting p53 K382 acetylation (K382ac), which mediates p53 functional activation and enhanced apoptosis following DNA damage [16] (Figure 2B). In colorectal cancer, ArhGAP30 is frequently downregulated, promoting tumor cell proliferation, migration, and apoptosis inhibition through p53-dependent mechanisms [16].

In 1977, Goldknopf et al. discovered the ubiquitination modification of proteins [17]. Subsequently, the ubiquitin-mediated proteolytic pathway model was proposed in 1980 [18]. Ubiquitin is an evolutionarily conserved protein that labels substrate proteins through an enzymatic cascade mediated by ubiquitin activating enzymes (E1), ubiquitin conjugating enzymes (E2), and ubiquitin ligases (E3), resulting in ubiquitination [19]. Comprising 76 amino acids, ubiquitin contains seven lysine residues, all of which can undergo ubiquitination to form ubiquitin chains [20]. The most common type is the K48-linked ubiquitin chain, which primarily marks proteins for degradation by the proteasome, the second most common type is the K63-linked ubiquitin chain, which performs various non-degradative functions [20].In prostate cancer, DTX3L promotes the ubiquitination and degradation of TIRR, which impairs TIRR’s negative regulation of 53BP1 [21]. The interaction of 53BP1 with double-strand break (DSB) sites on chromatin leads to homologous recombination (HR) deficiency and enhances tumor sensitivity to PARP inhibitors [21]. Ubiquitination does not always direct proteins to proteasomal degradation. SPOP promotes K27-linked non-degradative polyubiquitination of Geminin at K100 and K127, which plays an oncogenic role in preventing DNA replication overactivation by indirectly blocking Cdt1-MCM complex interaction [22] (Figure 2C). Ubiquitination regulates diverse cellular processes through both degradative and non-degradative mechanisms, playing critical roles in tumorigenesis and progression [23].

Ubiquitination marks the emergence of small polypeptide-mediated PTMs in proteins. Notably, eukaryotes have evolved into a family of ubiquitin-like proteins (UBLs) that are structurally homologous to ubiquitin but functionally distinct. Phylogenetically, UBLs encompass nine different categories, including SUMO, NEDD8, ISG15, FUB1, FAT10, Atg8, Atg12, Urm1, and Ufm1 [24,25]. The wave of discoveries regarding their modification systems occurred predominantly from the 1980s to the early 2000s [24]. Similar to ubiquitination, UBL modifications involve a three-step enzymatic cascade mediated by E1, E2, and E3, which covalently attach single UBL or UBL chains to lysine residues on target proteins [26,27]. Among them, SUMOylation and neddylation are the most extensively studied UBL modification systems and recent studies have underscored their roles in tumor-associated processes [27,28].

Since the 21st century, a diverse array of lysine modifications, including propionylation [29], butyrylation [29], succinylation [5], malonylation [30], crotonylation [31], glutarylation [32], hydroxyisobutyrylation [33], β-hydroxybutyrylation [34], and lactylation [35], has been progressively characterized. Lactylation is a representative of newly discovered lysine PTMs, initially reported by Zhang et al. in 2019 [35]. Lactate condenses with CoA in the cytoplasm to generate lactyl-CoA, which acts as the lactyl donor for lactylation [36]. Early studies identified “writers” of protein lactylation, such as p300, increase the levels of lactylation through their general acyl transfer activity [37]. Recent studies have brought new advances in lactylation research, including the identification of ACSS2 as the bona fide lactyl-CoA synthetase in mammals and AARS1/2 as pan-lactyltransferases [38,39]. CBP-induced lactylation of the K673 site of MRE11 enhances its DNA-binding ability, strengthens DNA resection, and promotes HR repair, leading to chemoresistance in colon cancer cells [40]. Another study shows that in gastric cancer, TIP60-induced lactylation of the K388 site of NBS1 promotes its formation of a trimeric MRN complex with MRE11 and RAD50, thereby activating subsequent DSB sensing and DNA repair pathways [41] (Figure 2D). By modifying non-histone proteins, lactylation exerts diverse effects on tumors [42].

Moreover, other PTMs can also occur on non-histone lysine residues and have been preliminarily validated for their roles in tumor biology, such as succinylation, crotonylation, malonylation, β-hydroxybutyrylation and glutarylation [43].

### 2.2. Regulation of Tumor-Associated Proteins Through Non-Histone Lysine PTMs

p53 is a pivotal tumor suppressor that inhibits tumorigenesis and growth through both transcription-dependent and transcription-independent mechanisms [44]. Notably, p53 was the first non-histone protein identified to be regulated by lysine methylation. SETD7 catalyzes monomethylation of p53, which enhances its stability and promotes the expression of its target genes [45]. In contrast, SMYD2-mediated methylation at K370 suppresses p53 transcriptional activity [46]. Acetylation affects the transcriptional activity of p53. CBP/p300 acetylates p53 at multiple lysine residues (K370, K372, K373, K381, K382), thereby activating its sequence-specific DNA binding and transcriptional activity [47,48]. Conversely, HDAC1 and SIRT1 bind to and deacetylate p53, suppressing p53-dependent gene transcription and apoptosis in response to DNA damage and oxidative stress [49,50,51].

MYC, as one of the most prominent oncoproteins, is overexpressed in most malignancies [52]. HDAC1-mediated deacetylation of MYC K148, promoting MYC deubiquitination and stabilization [53]. Another study identified that HDAC3-mediated deacetylation at K148 selectively modulates MYC transcriptional activity without affecting its protein stability [54]. Bladder cancer employs a multilayer PTM network to cooperatively maintain the stability of oncogenic MYC [55]. Mechanistically, SETD8 catalyzes methylation of MYC K412, directly preventing interaction with the E3 ubiquitin ligase CHIP and ubiquitination-dependent degradation; simultaneously, SUMOylation stabilizes SETD8 and enhances its binding efficiency to MYC, further boosting MYC methylation levels and protein stability [55].

HIF1α plays a critical role in many aspects of cancer biology. As a result of intratumoral hypoxia, HIF1α activity is frequently heightened, promoting glycolysis and lactate production [56]. Tumor lactate accumulation also promotes angiogenesis and cancer stemness by enhancing lactylation of HIF1α [57]. SETD7/9 methylates HIF1α, promoting its degradation, whereas the demethylase activity of LSD1 enhances HIF1α stability and contributes to tumor angiogenesis [58,59] PPA2 recruits the E3 ligase NEDD4 to degrade HIF-1α, while PPA2 desuccinylation inhibits NEDD4 activation, promoting HIF-1α stability and glycolysis [60].

Although our knowledge of non-histone lysine PTMs continues to expand, their physiological significance remains largely uncharacterized. Global proteomic analyses have revealed that the regulatory scope of PTMs is far greater than previously recognized [61,62]. Consequently, key PTM substrates remain to be identified. Furthermore, while dysregulated PTMs are recognized as important drivers of tumor progression, more research is needed to explain the mechanisms underlying PTM imbalance in specific cancers. Tumor metabolic reprogramming may be a key contributor, as various metabolites have been shown to covalently modify proteins through lysine acylation [43].

## 3. Crosstalk of Non-Histone Lysine PTMs in Tumors

Different PTMs on proteins can regulate each other, known as PTMs crosstalk. Multiple PTMs on the same or on different proteins create combinatorial effects that collectively influence protein function. PTMs crosstalk expands the mechanisms of protein functional regulation and increases the information capacity of the proteome [63].

### 3.1. Competitive Occupancy of Different PTMs at Identical Lysine Sites

**Figure 3:**
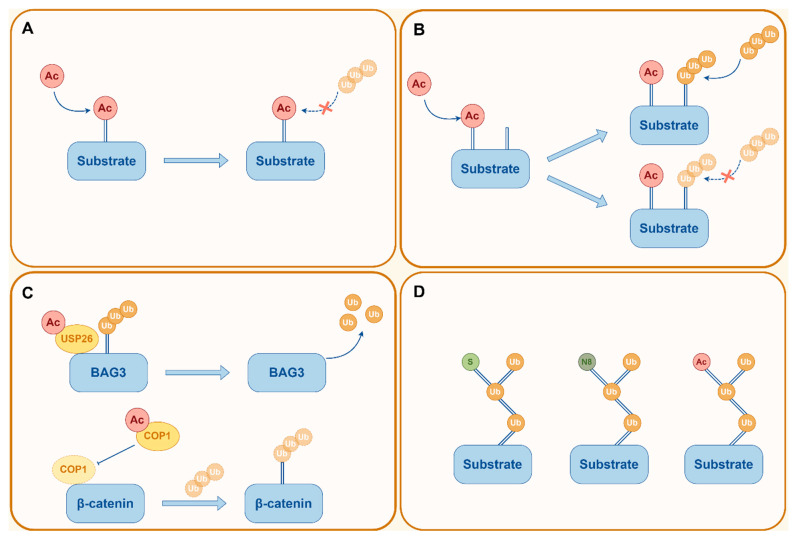
Crosstalk of lysine PTMs. Using acetylation and ubiquitination as examples, this figure illustrates four patterns of PTM crosstalk. (**A**) Competitive occupancy of different PTMs at identical lysine sites. (**B**) A modification at one site facilitates or inhibits modifications at neighboring sites. (**C**) PTMs occurring on modifying enzymes enhance or suppress their activity. (**D**) Hierarchical ubiquitin modifications, including SUMOylated ubiquitin, NEDDylated ubiquitin, and acetylated ubiquitin.

The competition between non-histone acetylation and ubiquitination at identical lysine residues to inhibit proteasome-dependent degradation of proteins represents a well-documented regulatory mechanism. For example, SEPT2 balances acetylation and ubiquitination of HSPA5 at K327, thereby alleviating endoplasmic reticulum stress and limiting macrophage M1 polarization [64]. In gastric cancer, YAP lactylation not only inhibits ubiquitination at the same site through competitive occupancy but also impedes its cytoplasmic translocation for ubiquitination [65]. A recent study revealed that the dynamic transition between crotonylation and SUMOylation at Ku80 K568 promotes resistance to radiotherapy in cancer [66]. Specifically, Ku80 K568 is crotonylated by PCAF and decrotonylated by HDAC8 upon DNA damage, priming this site for subsequent SUMOylation by CBX4 [66]. The shift from crotonylation to SUMOylation on Ku80 facilitates DNA-PK complex assembly and autophosphorylation, activating NHEJ repair to counteract DNA damage (Figure 3A).

### 3.2. Interdependence of Different PTMs at Neighboring Modification Sites

Interdependence between PTM sites within proximal sequences is a common mode of PTM crosstalk, which can exert either positive or negative effects [63]. For instance, acetylation at K311 serves as a prerequisite for the interaction between GAC and TRIM21, which suppresses GAC functionality by facilitating K63-linked ubiquitination, thereby contributing to the suppression of lung carcinogenesis [67]. Conversely, LONP1 K145 acetylation inhibits ubiquitin binding, while the LONP1 K145R mutant exhibits strong affinity to K63 ubiquitin [68]. SIRT3-mediated deacetylation restricts tumor initiation by promoting LONP1 ubiquitination and degradation [68]. Beyond the interactions between acetylation and ubiquitination, this crosstalk mechanism also occurs among other lysine PTMs. N4-acetylcytidine, the sole conserved acetylation event in eukaryotic RNA, is catalyzed by NAT10, expanding the regulatory repertoire in RNA structure and function [69]. The 2-hydroxyisobutyrylation at K823 of NAT10 facilitates its physical interaction with USP39 and subsequent deubiquitination at K195 and K426, which stabilizes NAT10 protein levels [70]. Aberrant upregulation of NAT10 in tumor tissues promotes esophageal cancer metastasis by enhancing NOTCH3 mRNA stability in an N4-acetylcytidine-dependent manner [70] (Figure 3B).

### 3.3. Regulating the Activity of Modifying Enzymes Through PTMs

As pivotal regulators of PTMs, modifying enzymes’ lysine residues can also undergo various chemical modifications. For instance, ubiquitin-modifying enzymes can serve as substrates for acetylation. In breast cancer, Tip60-mediated acetylation of USP26 at K134 enhances its deubiquitinating activity toward BAG3, promoting tumor cell proliferation and invasion [71]. Conversely, in ovarian cancer, COP1 K294 acetylation impairs its function as an E3 ubiquitin ligase, resulting in β-catenin accumulation and enhanced activity [72]. Bladder cancer employs a multilayer PTM network to cooperatively maintain the stability of oncogenic MYC, driving tumor progression [55]. Mechanistically, SETD8 catalyzes methylation of MYC at K412, directly preventing interaction with the E3 ubiquitin ligase CHIP and ubiquitination-dependent degradation; simultaneously, SUMOylation stabilizes SETD8 and enhances its binding efficiency to MYC, further boosting MYC methylation levels and protein stability [55] (Figure 3C).

### 3.4. Hierarchical Modification of Ubiquitination

As a protein containing seven lysine residues, ubiquitin forms polyubiquitin chains with specific topological structures through lysine-linked ubiquitylation, establishing a comprehensive ubiquitin code. Given that ubiquitin itself can undergo ubiquitination, investigations have explored other potential PTMs on ubiquitin and reported acetylation, SUMOylation, and neddylation. Acetylation occupies functional lysine residues on ubiquitin, thereby interfering with chain assembly. Initial studies detected acetylation at ubiquitin K6 or K48 in cellular contexts, a modification that does not impair substrate monoubiquitination but inhibits E2 enzyme-dependent elongation of K11-, K48-, and K63-linked polyubiquitin chains [73]. Notably, HDAC inhibitor (HDACi) treatment upregulates ubiquitin acetylation levels in the non-small cell lung cancer cell line A549 [74]. In HeLa cells exposed to ionizing radiation, acetylation events were observed at six lysine residues of ubiquitin except K29, suggesting potential roles of ubiquitin acetylation in DDR signaling [75]. SUMO can also conjugate to ubiquitin via lysine residues (K6, K11, K27, K48, and K63), with increased K6- and K27-linked SUMO-modified ubiquitin chains under conditions of heat shock or proteasome inhibition [20]. As the closest UBL to ubiquitin, Nedd8 forms hybrid chains with ubiquitin and acts as a chain terminator [76]. Interestingly, proteins marked by K48-linked Neddylated ubiquitin chains are still recognized and degraded by the proteasome [76]. Collectively, the addition of auxiliary PTMs to ubiquitin introduces an additional layer of complexity to the ubiquitin code. The biological functions underlying these crosstalk mechanisms remain poorly understood, highlighting new directions for investigating the interplay between PTMs (Figure 3D).

## 4. Non-Histone Lysine PTMs in TIME

### 4.1. Non-Histone Lysine PTMs in T Cells

**Figure 4:**
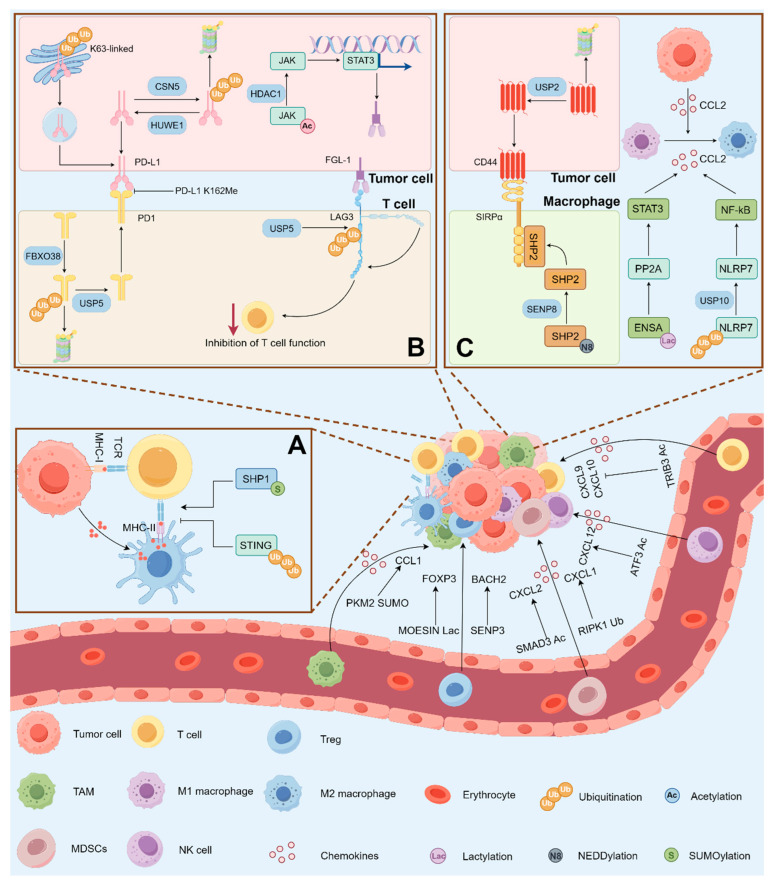
Non-histone lysine PTMs in tumor immune microenvironment. Various immune cells are recruited to the tumor bed by chemokines, such as T cells (CXCL9, CXCL10), macrophages (CCL2), NK cells (CXCL12), and MDSCs (CXCL1, CXCL2). The secretion of these chemokines is regulated by non-histone acetylation, ubiquitination, and SUMOylation, consequently impacting immune cell abundance within the TME. Beyond this, non-histone PTMs also modulate immune cell functionality. (**A**) SHP1 SUMOylation and STING ubiquitination influence the antigen-presenting function of DCs. (**B**) The PD-1/PD-L1 signaling axis, critical for T cell cytotoxic function, is regulated by multiple PTMs including methylation, acetylation, and ubiquitination. (**C**) The CD47/SIRPα axis, which governs macrophage phagocytic function, is modulated by ubiquitination and SUMOylation. ENSA lactylation and NLRP7 deubiquitination promote M2 macrophage polarization via enhanced CCL2 secretion.

CD8^+^ T cells represent the most potent effectors in cellular immunity, directly recognizing and eliminating cancer cells [77]. CXCR3 expressed on CD8^+^ effector T cells mediates tumor-directed migration in response to CXCL9 and CXCL10 chemokines [78]. Acetylation enhances TRIB3 stability, inhibiting T cell infiltration by suppressing the STAT1/CXCL10 axis, thereby promoting immune evasion in colorectal cancer [79]. Pharmacological inhibition of P300 enhances TRIB3 degradation via reduced acetylation, sensitizing tumors to immune checkpoint inhibitors (ICIs) treatment [79]. K63 ubiquitination of STING at K224 facilitates the binding of autophagy receptor TAX1BP1 to STING, leading to proteasomal degradation [80]. Furthermore, combined treatment with ATR inhibitors and irradiation induces SHP1 SUMOylation, activating non-canonical STING signaling by diminishing SHP1-mediated inhibition of the TRAF6/STING/p65 axis [81]. STING activation enhances T cell infiltration, transforming “cold” tumors into “hot” tumors, thus promoting antitumor immunity. (Figure 4A).

Multiple inhibitory receptors, including PD-1, LAG3, and TIM3, are expressed on T cell surfaces to protect healthy tissues from damage caused by hyperactivated immune responses [82]. Tumors, however, exploit coinhibitory pathways by upregulating corresponding ligands of these receptors, thereby mediating immunosuppressive effects [82]. The expression and activity of PD-1/PD-L1 are modulated by diverse PTMs, which critically influence T cell-mediated immunity. Surface PD-1/PD-L1 molecules can be internalized, ubiquitinated, and degraded by proteasomes, while tumors often suppress degradation-associated ubiquitination to stabilize PD-1/PD-L1 and deliver coinhibitory signals to T cells [83,84,85,86]. MIB2 can also catalyze nonproteolytic K63-linked ubiquitination of PD-L1, promoting its trafficking from the trans-Golgi network to the plasma membrane of cancer cells [87]. Independent of its immunosuppressive function at the plasma membrane, deacetylation at K263 facilitates nuclear accumulation of PD-L1 and triggers the expression of additional immune checkpoint molecules [88]. Inhibition of HDAC2 to block PD-L1 nuclear entry reduces these checkpoint genes expression and enhances CD8^+^ cytotoxic T cell infiltration in tumors [88]. In contrast, methylation at K162 does not alter PD-L1 expression, stability, or membrane translocation but instead constrains PD-L1/PD-1 complex formation, thereby potentiating T cell-mediated tumor elimination [89]. PTMs on immune checkpoints provide new opportunities for targeted therapy. Using deubiquitinase inhibitors can promote the degradation of PD-L1 and enhance the efficacy of immune ICIs [90,91,92]. CPT1A-mediated succinylation of PD-L1 also facilitates its degradation. The hyperlipidemic drug bezafibrate upregulates CPT1A and synergizes with anti-PD-L1 therapy to inhibit tumor growth [93]. LAG3 represents another promising target in cancer immunotherapy. A recent study revealed that non-degradative ubiquitination of LAG3 K498 is crucial for its immunosuppressive function [94]. Several LAG3-targeting agents significantly inhibit this ubiquitination, and the ability of these antibodies to suppress LAG3 ubiquitination strongly correlates with enhanced IL-2 production [94]. Another study demonstrated that acetylation promotes JAK1 degradation and reduces STAT3-driven FGL1 transcription, combination therapy with an HDAC1 inhibitor and anti-LAG3 further enhanced the cytotoxic activity of CD8^+^ T cells [95]. (Figure 4B).

BCAA metabolism regulates T cell proliferation, differentiation, and functionality [96]. SIRT7 deficiency in T cells elevates succinylation levels of key enzymes in BCAA catabolic pathways, resulting in accumulation of BCAA metabolites and fatty acids, diminished IFNγ secretion, and induction of T cell exhaustion [97]. As a cytotoxic effector molecule of CD8^+^ T cells, IFNγ downregulates expression of SLC3A2 and SLC7A11, two subunits of glutamate-cystine antiporter system xc-, rendering tumor cells more susceptible to ferroptosis [98]. IL-1β induces mitochondrial translocation of PCAF and subsequent NNT K1042ac, promoting NADPH production and cellular reductive capacity to protect tumors from T cell-driven ferroptosis [99]. Anti-IL-1β antibody suppresses NNT acetylation, thereby sensitizing tumors to checkpoint therapy [99].

### 4.2. Non-Histone Lysine PTMs in Macrophages

Macrophages, essential components of innate immunity, perform diverse functions including phagocytosis, antigen presentation, and cytokine secretion [100]. Based on functional states under distinct stimuli, macrophages are categorized into classically activated M1 (pro-inflammatory) and alternatively activated M2 (anti-inflammatory) subtypes, with tumor-associated macrophages (TAMs) predominantly exhibiting an M2-like phenotype [101]. The surface receptors and secreted mediators of TAMs play multiple roles in tumor development and progression by supporting tumor growth, inhibiting apoptosis, promoting angiogenesis, and disrupting immune surveillance [100]. Circulating monocytes are the primary source of TAMs, which are recruited and stimulated by tumor-derived growth factors and chemokines to migrate into tumors and differentiate into macrophages [102]. In hepatocellular carcinoma cells, SUMOylation promotes PKM2 sorting into extracellular vesicles in an ARRDC1-dependent manner, inducing monocyte differentiation into TAMs via intercellular communication [103]. Meanwhile, cytokines secreted by macrophages enhance PKM2 excretion through the CCL1/CCR8 axis, forming a feed-forward regulatory loop that promotes tumorigenesis [103].

As the predominant phagocytic population in tumors, TAM phagocytic capacity is regulated by the CD47/SIRPα axis, which transmits “don’t eat me” signals [104]. USP2 deubiquitinates and positively regulates CD47 abundance, whereas USP2 inhibition enhances macrophage phagocytosis and remodels the immunosuppressive TME [105]. Upon recognition of CD47 on target cells, macrophage SIRPα triggers phosphorylation of its cytoplasmic region immunoreceptor tyrosine-based inhibitory motif, followed by recruitment of SHP1 and SHP2 for signal transduction [106]. Neddylation at SHP2 K358 and K364 inhibits its activity, thereby blocking the phagocytic inhibitory signals of CD47/SIRPα, promoting phagocytosis and clearance of tumor cells by macrophages [107]. Emerging perspectives highlight that macrophages can perform antigen cross-presentation via both cytosolic and vacuolar pathways, aiding in the activation of CD8^+^ T cells similarly to dendritic cells (DCs) [108]. In cholangiocarcinoma, PDHA1 K83 succinylation leads to α-ketoglutarate accumulation in TME, triggering MAPK signaling through OXGR1 receptors on macrophages and suppressing MHC-II antigen presentation, resulting in immune evasion and tumor progression [109]. (Figure 4C).

CCL2 and CSF-1 are among the most critical factors for macrophage recruitment and M2 polarization, with CCL2 inducing differentiation of macrophages toward a pro-tumoral phenotype through its cell surface receptor CCR2 [110]. CCL2 has been identified as a target gene of the oncogenic transcription factors NF-κB and STAT3 [111,112]. USP10 deubiquitinates and stabilizes NLRP7 protein, promoting CCL2 secretion and M2-like macrophage polarization through NF-κB signaling activation [113].

### 4.3. Non-Histone Lysine PTMs in Other Immune Cells

Intratumoral regulatory T cells (Tregs) suppress effector T cell function and serve as major contributors to tumor immune tolerance [114]. Tumor-infiltrating Tregs exhibit elevated SENP3 expression, which maintains their immunoregulatory capacity by mediating BACH2 deSUMOylation to prevent nuclear export [115].

Myeloid-derived suppressor cells (MDSCs), another subset of immunosuppressive regulators in TME, are recruited to tumor beds through CXCL1- and CXCL2-dependent chemotaxis [116]. TRIM28 promotes K63-linked polyubiquitination of RIPK1 to activate the NF-κB signaling pathway, which induces potent chemokine CXCL1 production and amplifies MDSC infiltration [117]. Similarly, KAT6A-catalyzed acetylation strengthens SMAD3 binding to the CXCL2 promoter, facilitating MDSC recruitment and tumor metastasis [118].

Natural killer (NK) cells are the primary effector cells in antitumor innate immunity and have emerged as promising therapeutic targets in cancer treatment [119]. CXCL12 secretion stimulates NK cell recruitment, while HDAC3-mediated deacetylation of ATF3 suppresses NF-κB transcriptional activity, thereby downregulating CXCL12 expression [120]. Pharmacological inhibition of HDAC3 enhances NK cell infiltration and improves tumor response to conventional chemotherapy [120].

### 4.4. Lactate and Non-Histone Lactylation in TIME

Enhanced glycolytic activity in tumors fosters the formation of an immunosuppressive TIME through lactate and lactylation. Lactate impairs the antigen-presenting function of DCs, thereby hindering T cell activation [121]. Furthermore, lactate suppresses T cell function by inhibiting the activation of the p38 and JNK/c-Jun pathways [122]. Additionally, a highly glycolytic TME upregulates PD-1 expression in Tregs, contributing to immunotherapy failure [123]. Beyond its direct effects, lactate also suppresses anti-tumor immunity via non-histone lysine lactylation. Lactylation of ENSA promotes the expression of CCL2, leading to the enrichment of immunosuppressive macrophages [124]. MOESIN K27 lactylation induces Treg differentiation by promoting FOXP3 expression [125]. Moreover, lactylation enhances the activity of APOC2, driving the accumulation of free fatty acids (FFA) in the TME, which subsequently promotes the accumulation of Tregs [126].

Non-histone lysine PTMs influence the infiltration and function of immune cells, contributing to the formation of an immunosuppressive TIME (Table 1). Particularly, PTMs impact the TIME and the efficacy of ICIs by regulating the expression and function of immune checkpoint molecules [127]. Current research is primarily focused on the PTMs of classic molecules like PD-1 and PD-L1, while the understanding of PTM-mediated regulation of newer checkpoints such as LAG-3, TIM-3, and TIGIT remains limited. Furthermore, targeting the PTMs of immune checkpoints represents a promising strategy to improve immunotherapy responses, future research should focus more on the development and application of such agents.

## 5. Therapeutic Strategies Targeting Lysine PTMs

### 5.1. Targeting Substrates and Their Upstream/Downstream Pathways

The role of dysregulated PTMs on protein lysine residues in tumor progression is unquestionable. The biological effects of non-histone lysine modifications are determined by the function of their substrate proteins, making targeting of these substrates and their upstream/downstream pathways a viable therapeutic approach. For instance, the E3 ubiquitin ligase FBXL6 promotes tumor metastasis through the KRAS/MEK/ERK/mTOR pathway, and this effect is significantly blocked by the mTOR inhibitor everolimus and the MEK inhibitor trametinib [128]. While most studies focus on PTM-mediated functional alterations in individual proteins, accumulating evidence suggests that PTMs can coordinately regulate multiple proteins within specific biological processes. In the case of autophagy, acetylation promotes autophagy in cancer through modifications of PAK1 and FOXO1, an effect that can be reversed by inhibition of PAK1 or FOXO1 [129,130]. However, considering that PTMs exert their effects through the modulation of multiple pathways, targeting a single pathway may be associated with limited therapeutic efficacy.

### 5.2. Targeting Modifying Enzymes

A broader therapeutic approach involves targeting modifying enzymes. The therapeutic potential of targeting these enzymes was first demonstrated in the 1970 s when HDACi was shown to control leukemic cell differentiation by altering histone acetylation levels [131,132]. In 2006, SAHA became the first HDACi approved by the FDA for treating cutaneous T-cell lymphoma [133]. As an inhibitor of class I and II HDACs, SAHA exhibits multiple targets, including non-histone proteins linked to cell proliferation, migration, and death [133]. SIRTs, another major class of deacetylases, have also been identified as targets for cancer therapy, with small-molecule activators and inhibitors developed against them [134]. Agents targeting SIRTs exert anticancer effects partly through non-histone substrates such as PI3K, FOXQ1, and SMAD4 [135,136,137]. While several KDAC inhibitors are clinically approved, drugs targeting KATs remain distant from clinical application [138]. For methylation, the most extensively studied inhibitors in clinical trials target EZH2 and DOT1L, which mediate H3K27 and H3K79 methylation, respectively [139]. Beyond EZH2 and DOT1L, selective inhibitors of other KMTs, including SETD7, SETD8, SMYD2, and SMYD3, are under preclinical evaluation [139]. Notably, these KMTs also methylate non-histone proteins such as p53 [140]. Multiple drugs targeting the ubiquitination system, particularly the E3 ubiquitin ligase MDM2, have completed Phase I/II trials for hematologic malignancies and solid tumors [23]. In addition to inhibiting p53, MDM2 contributes to tumor initiation, evasion of cell death, metastasis, and chemoresistance, making it an attractive target in cancer therapy [141]. Hyperactivation of SUMOylation and neddylation pathways has been observed in various human cancers, and targeting the enzymes involved in these processes also offers a promising antitumor strategy [27,28] (Table 2).

### 5.3. Dietary Interventions

**Figure 5:**
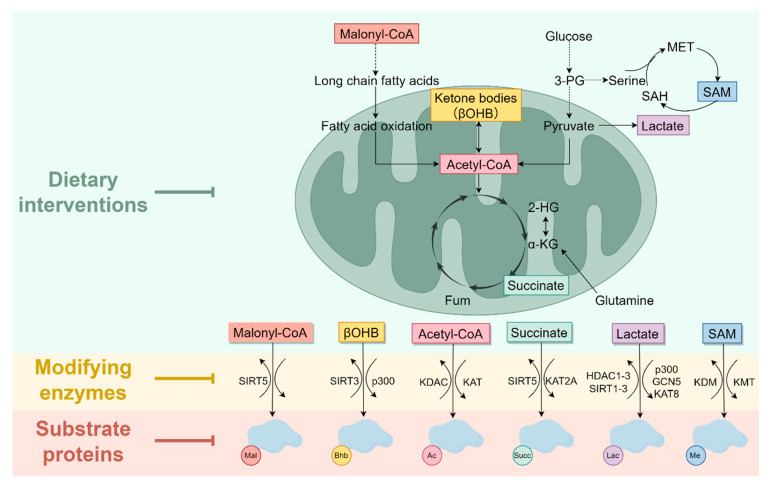
Therapeutic strategies targeting lysine PTMs. Dietary patterns can influence the abundance of cellular metabolites. Metabolites serve as acyl donors and participate in lysine PTMs, such as methylation, acetylation, lactylation, and succinylation, under the catalysis of modifying enzymes. Consequently, therapeutic strategies encompass dietary intervention, targeting modifying enzymes, and targeting substrates and their upstream/downstream pathways.

Diet and lifestyle represent critical determinants of cancer risk, with fasting, calorie restriction, and ketogenic diets inducing broad alterations in growth factor and metabolite levels to potentiate cancer treatment efficacy [142]. The ketogenic diet promotes fatty acids β-oxidation and ketone bodies production, generating Ac-CoA and BHB as the respective acyl donors for lysine acetylation and β-hydroxybutyrylation [143]. BHB supplementation elevates β-hydroxybutyrylation at ALDOB K108, suppressing its enzymatic activity [144]. Experimental evidence demonstrates that ALDOB β-hydroxybutyrylation significantly inhibits proliferation across hepatocellular, renal, and gastric cancers by blocking mTOR signaling and glycolysis [144]. In addition, BHB is an endogenous and selective class I HDACs inhibitor that also enhances global protein acetylation [145]. Serine and glycine are exploited by numerous cancers to sustain progression, particularly for survival and proliferation under nutrient-deprived conditions [146]. A serine/glycine-free diet (−SG diet) has been shown to be effective in inhibiting tumor growth [147,148]. While the −SG diet induces tumor cell secretion of chemokines such as CCL5 and CXCL11 to promote CD8^+^ T cell recruitment, it concurrently increases tumor lactate production, which stabilizes PD-L1 via lactylation [149]. Therefore, combining the −SG diet with anti-PD-1/PD-L1 therapy is necessary. These findings informed a single-arm Phase I trial confirming the safety and feasibility of −SG diets in advanced solid tumor patients [149]. AARS1/2, initially classified as aminoacyl-tRNA synthetases for catalyzing the attachment of amino acids to their corresponding tRNAs, function as lactyltransferases mediating lactylation of non-histone substrates including p53 and cGAS [37]. Given their dual roles in protein translation and lactylation, studies have judiciously employed β-alanine and L-alanine supplementation to competitively inhibit lactate-AARS1/2 binding for anticancer effects [39,65,150]. Incidentally, a recent intriguing study uncovered that vitamin C directly modifies lysine residues, termed Vitcylation [151]. This work demonstrated that Vitcylation of STAT1 at lysine 298 enhances IFNγ signaling and activates antitumor immunity, shedding new light on the anticancer mechanism of dietary vitamin C [151]. In summary, dietary-based approaches represent a valuable strategy for cancer treatment (Figure 5).

### 5.4. Challenges and Future Perspectives

Although drugs targeting PTMs show broad prospects in cancer treatment, they still face several challenges. A major obstacle is the limited specificity of currently developed drugs. Inhibitors targeting conserved catalytic sites within enzyme families often cause off-target effects and toxicity [26,152]. Developing inhibitors with higher selectivity may enhance therapeutic efficacy while reducing adverse reactions in the future. Furthermore, the activation of compensatory signaling pathways in tumors can lead to resistance against targeted monotherapy, making single-pathway inhibitors highly limited [153]. Combination therapy may enhance treatment effects and overcome drug resistance. For instance, cancer patients who are unresponsive to HDACi often show upregulation of CD47, which promotes macrophage polarization to the M2 phenotype [154]. Combining HDACi with CD47 neutralizing antibody can reverse resistance to HDACi. Future efforts should focus on translating novel therapeutic strategies into clinical practice. Monoclonal antibodies or small molecules that specifically target PTM sites may serve as alternative options for cancer treatment [155]. Proteolysis-targeting chimeras (PROTACs), which co-opt UPS to selectively target cellular proteins for durable degradation, have emerged as a new modality for precision medicine [156]. By overcoming the limitations of traditional small-molecule inhibitors, PROTACs offer the potential to target the undruggable proteins.

**Table 2:** Inhibitors targeting PTMs for cancer therapy.

Targets	Cancer	Treatment	Phases	Study Status	NCT Number
Targeting methylation for cancer therapy					
EZH2	Non-Hodgkin Lymphoma and Solid Tumor	AXT-1003	Phase1	Recruiting	NCT06484985
	Non-Hodgkin Lymphoma	AXT-1003	Phase1	Terminated	NCT05965505
	Ovarian Cancer	CPI-0209	Phase1	Recruiting	NCT05942300
	Peripheral T Cell Lymphoma	SHR2554	Phase1	Not Yet Recruiting	NCT06712173
	Solid Tumor and Lymphoma	SHR2554	Phase1/Phase2	Recruiting	NCT04407741
	Lymphoma	Tazemetostat	Phase2	Recruiting	NCT06692452
	Lymphoma	Tazemetostat	Phase2	Not Yet Recruiting	NCT06068881
	Follicular Lymphoma	Tazemetostat	Phase1/Phase2	Recruiting	NCT05551936
	Peripheral Nerve Sheath Tumor	Tazemetostat	Phase2	Active Not Recruiting	NCT04917042
	Melanoma	Tazemetostat	Phase1/Phase2	Active Not Recruiting	NCT04557956
	Lymphoma	Tazemetostat	Phase3	Recruiting	NCT04224493
	Non-Small Cell Lung Cancer	Tulmimetostat	Phase1/Phase2	Not Yet Recruiting	NCT05467748
	Advanced Tumor and Lymphoma	XNW5004	Phase1/Phase2	Recruiting	NCT06558513
	Solid Tumors	XNW5004	Phase1/Phase2	Recruiting	NCT06022757
DOT1L	Acute Myeloid Leukemia	Pinometostat	Phase1/Phase2	Completed	NCT03701295
Targeting acetylation for cancer therapy					
HDAC	Colon Adenocarcinoma	Chidamide	Phase2	Recruiting	NCT06709885
	Peripheral T-cell Lymphoma	Chidamide	Phase1/Phase2	Recruiting	NCT06421948
	Acute Lymphoblastic Leukemia	Chidamide	Phase2	Recruiting	NCT06220487
	Metastatic Colorectal Cancer	Sodium Valproate	Phase2	Recruiting	NCT05694936
	Breast Cancer	chidamide	Phase2	Unknown	NCT05438706
	Non-Hodgkin Lymphoma and Solid Tumor	Chidamide	Phase1/Phase2	Unknown	NCT05320640
	Neuroendocrine Tumor	Chidamide	Phase2	Unknown	NCT05113355
	Neuroendocrine Tumor	Chidamide	Phase2	Unknown	NCT05076786
	Solid Tumors and Lymphoma	Entinostat	Phase1/Phase2	Recruiting	NCT05053971
	Cervical Cancer	Chidamide	Phase1/Phase2	Unknown	NCT04651127
	T-cell Leukemia	Romidepsin	Phase1	Withdrawn	NCT04639843
	Non-Small Cell Lung Cancer	Entinostat	Phase1	Completed	NCT04631029
	Non-Hodgkin Lymphoma	Chidamide	Phase1/Phase2	Unknown	NCT04553393
	Hodgkin Lymphoma	Chidamide	Phase2	Unknown	NCT04514081
	Peripheral T-cell Lymphoma	Chidamide	Phase2	Recruiting	NCT04512534
	Non-Hodgkin Lymphoma	Chidamide	Phase1/Phase2	Recruiting	NCT04337606
	Breast Cancer	Entinostat	Phase1	Terminated	NCT04296942
	Hodgkin Lymphoma	Chidamide	Phase2	Recruiting	NCT04233294
	Lymphoma and Follicular	Abexinostat	Phase2	Active Not Recruiting	NCT03934567
	Solid Tumor and Lymphoma	Entinostat	Phase1	Withdrawn	NCT03925428
	Melanoma	Vorinostat	EARLYPhase1	Withdrawn	NCT03022565
KAT6A/B	Solid Tumors	OP-3136	Phase1	Recruiting	NCT06784193
Targeting ubiquitination for cancer therapy					
MDM2	Acute Myeloid Leukemia	Navtemadlin	Phase1	Active Not Recruiting	NCT04190550
	Acute Myeloid Leukemia	Siremadlin	Phase1/Phase2	Terminated	NCT05447663
	Acute Myeloid Leukemia	Siremadlin	Phase1	Terminated	NCT05155709
	Acute Myeloid Leukemia	Siremadlin	Phase1	Terminated	NCT04496999
	Soft-tissue Sarcoma	Siremadlin	Phase1/Phase2	Recruiting	NCT05180695
	Solid Tumor	Siremadlin	Phase2	Recruiting	NCT04116541
	Solid Tumor	Milademetan	Phase1/Phase2	Withdrawn	NCT06090318
	Solid Tumor	Milademetan	Phase2	Terminated	NCT05012397
	Dedifferentiated Liposarcoma	Milademetan	Phase3	Terminated	NCT04979442
	Breast Cancer	Milademetan	Phase2	Terminated	NCT05932667
	Neuroblastoma	APG-2575	Phase1	Recruiting	NCT05701306
	Liposarcoma	APG-2575	Phase1/Phase2	Recruiting	NCT04785196
	Leukemia	APG-2575	Phase1/Phase2	Recruiting	NCT04358393
	Leukemia	APG-2575	Phase1	Recruiting	NCT04275518
CUL4-DDB1-CRBM-RBX1 E3 complex	Acute Myeloid Leukemia	CC-90009	Phase1	Terminated	NCT04336982
CBL-B	Advanced Malignancies	NX-1607	Phase1	Recruiting	NCT05107674
CRL4	Hematological Malignancies	KPG-818	Phase1	Completed	NCT04283097
USP1	Solid Tumor	KSQ-4279	Phase1	Active Not Recruiting	NCT05240898
Targeting SUMOylation for cancer therapy					
SUMO-activating enzyme	Head and Neck Cancer	TAK-981	EARLYPhase1	Completed	NCT04065555
	Solid Tumors	TAK-981	Phase1/Phase2	Completed	NCT04381650
	Solid Tumors	TAK-981	Phase1	Terminated	NCT05976334
Targeting neddylation for cancer therapy					
NEDD8-activating enzyme	Acute Myelogenous Leukemia	Pevonedistat	Phase1	Terminated	NCT04172844
	Acute Myeloid Leukemia	Pevonedistat	Phase2	Completed	NCT04266795
	Acute Myeloid Leukemia and Myelodysplastic Syndromes	Pevonedistat	Phase2	Completed	NCT04712942
	Cholangiocarcinoma and Hepatocellular Carcinoma	Pevonedistat	Phase2	Active Not Recruiting	NCT04175912
	Multiple Myeloma	Pevonedistat	Phase1	Completed	NCT03770260
	Non-Small Cell Lung Cancer	Pevonedistat	Phase2	Active Not Recruiting	NCT03965689
	Solid Tumor	Pevonedistat	Phase1/Phase2	Terminated	NCT04800627

## 6. Conclusions and Future Perspectives

Dysregulated PTMs are important for tumorigenesis and progression, and focusing on non-histone substrates is a direction for PTMs research. Here, we comprehensively summarize the emerging mechanisms of non-histone PTMs in tumor microenvironment. Recent advancements in tumor research regarding PTMs have focused on the identification of novel modification types represented by lactylation. The widespread activation of the Warburg effect in tumors leading to lactate accumulation has made lactate an important focus in tumor biology research [157]. The discovery of protein lactylation provides an explanation for the significance of glycolytic activation in tumors from the perspective of PTMs, expanding the role of lactate beyond that of a mere energy fuel or signaling molecule [158].

With the continuous discovery of distinct modification types and sites, it is now a well-established fact that lysine residues on proteins are not exclusive targets of single PTMs. Multiple PTMs occurring on lysine residues form complex regulatory networks through synergistic or antagonistic interplay, extending far beyond the simplistic “one modification-one function” paradigm. Research on PTM crosstalk helps us better understand PTM codes and represents one of the major challenges for future studies [159].

The key role of PTMs in cancer has driven the exploration of therapeutic strategies targeting this mechanism. An exciting fact is that several drugs targeting PTMs have already been used in clinical treatment, and many more are currently in clinical trials. Although existing drugs have shown certain therapeutic effects, the development of other drugs still faces challenges [160]. In addition, identifying targets with high efficacy and low toxicity remains a key issue to be addressed. Interestingly, studies have shown that dietary interventions also exhibit anti-tumor effects by modulating PTM processes [161]. This may represent a simple and effective complementary therapy to drugs and surgery.

In summary, research on non-histone lysine PTMs has deepened our understanding of tumor pathogenesis. As research in this field continues to advance, it holds promise for providing new targets for precision cancer therapy.

## Figures and Tables

**Table 1:** Non-histone lysine PTMs in tumor immune microenvironment.

Immune Cells	PTMs	Target Protein	Sites	Writer	Eraser	Involved Pathways	Main Effects	References
T cell	Acetylation	TRIB3	K240	p300	-	P300/TRIB3/EGFR/STAT1/CXCL10	Enhance T cell infiltration	[79]
		PD-L1	K263	p300	HDAC2	P300-HDAC2/PD-L1	Inhibit the immune function of T cell	[88]
		JAK	K1109		HDAC1	HDAC1/JAK1/STAT3/FGL1	Inhibit the immune function of T cell	[95]
		NNT	K1042	PCAF	-	IL-1β/PCAF/NNT/NADPH	Inhibit the immune function of T cell	[99]
	Ubiquitination	STING1	K244	-	-	STING1/TAX1BP1	Enhance T cell infiltration	[80]
		PD-L1	-	TRIM28	-	TRIM28/TBK1/PD-L1	Inhibit the immune function of T cell	[86]
		LAG3	K498	c-Cbl, Cbl-b	-	FGL1/LAG3/c-Cbl, Cbl-b	Inhibit the immune function of T cell	[94]
	SUMOylation	SHP1	K127	-	-	SHP1/TRAF6-STING/p65	Enhance T cell infiltration	[81]
	Methylation	PD-L1	K162	SETD7	LSD2	SETD7-LSD2/PD-L1	Inhibit the immune function of T cell	[89]
Macrophage	SUMOylation	PKM2	-	UBC9	-	UBC9/PKM2/ARRDC1	Promote monocyte-to-TAM differentiation	[103]
	Ubiquitination	CD47	-	-	USP2	USP2/CD47/SIRPα	Enhance macrophage phagocytosis	[105]
		NLRP7	K379	-	USP10	USP10/NLRP7/NF-κB/CCL2	Promote M2 polarization of macrophages	[113]
	Neddylation	SHP2	K358, K364	-	SENP8	SENP8/SHP2/SIPRα	Enhance macrophage phagocytosis	[107]
	Succinylation	PDHA1	K83	DLST	-	PDHA1/α-KG/OXGR1/MAPK	Suppress macrophage antigen presentation	[109]
	Lactylation	ENSA	K63	P300	-	ENSA/PP2A/STAT3/CCL2	Promote M2 polarization of macrophages	[124]
Treg	Lactylation	MOESIN	K72	-	-	MOESIN/TGF-β/SMAD3	Promote Tregs accumulation	[125]
		APOC2	K70	P300	SIRT1, HDAC3	P300/APOC2/FFA	Promote Tregs accumulation	[126]
	Succinylation	BACH2	K172	-	SENP3	SENP3/BACH2	Promote Tregs accumulation	[115]
MDSC	Ubiquitination	RIPK1	-	TRIM28	-	TRIM28/RIPK1/NF-Κb/CXCL1	Enhance MDSCs infiltration	[117]
	Acetylation	SMAD3	K20, K117	KAT6A	-	KAT6A/SMAD3/TRIM24	Enhance MDSCs infiltration	[118]
NK cell	Acetylation	ATF3	K136, K139	-	HDAC3	HDAC3/ATF3/CXCL12	Enhance NK cells infiltration	[120]

## Data Availability

No datasets were generated or analyzed during the current study.

## Abbreviations

The following abbreviations are used in this manuscript:

PTMs	Post-translational modifications
TIME	tumor immune microenvironment
KMTs	lysine methyltransferases
KDMs	lysine demethylases
SAM	S-adenosylmethionine
KATs	lysine acetyltransferases
KDACs	lysine deacetylases
ASCC	activating signal cointegrator complex
DSB	double-strand break
HR	homologous recombination
UBLs	ubiquitin-like proteins
HDACi	HDAC inhibitor
ICIs	immune checkpoint inhibitors
TAMs	tumor-associated macrophages
Tregs	intratumoral regulatory T cells
MDSCs	myeloid-derived suppressor cells
NK cells	natural killer cells
FFA	free fatty acid
−SG diet	serine/glycine-free diet
PROTACs	proteolysis-targeting chimeras
